# Reply to “Comment on Neiser et al. Assessment of Dextran Antigenicity of Intravenous Iron Preparations with Enzyme-Linked Immunosorbent Assay (ELISA). *Int. J. Mol. Sci.* 2016, *17*, 1185.”

**DOI:** 10.3390/ijms18010122

**Published:** 2017-01-10

**Authors:** Susanna Burckhardt

**Affiliations:** Global Medical Affairs, Vifor Pharma Ltd., 8152 Glattbrugg, Switzerland; susanna.burckhardt@viforpharma.com; Tel.: +41-58-851-8000

## To the Editor:

Thank you providing the opportunity to reply to the Comment on our recent paper.

Strom and Andreasen, who are employees of Pharmacosmos, a Danish-based pharmaceutical company that commercializes iron dextran and iron isomaltoside 1000, criticize our work as it cannot be directly extrapolated to the clinical setting. However, as elucidated below, this was never the purpose of this scientific paper.

In response to Strom and Andreasen, I would like to state that, as clearly mentioned in the introduction, the experiments described in Neiser et al. [1] were triggered by the criticism [2] of our previous work published in 2011 [3]. Specifically, the *methodology* (reverse single radial immunodiffusion assay) and the use of *only one* specific anti-dextran antibody were criticized. Thus, the new experiments reported in [1] were carried out with a *new antibody* (produced by the service provider GenScript) and *a different technique* (Enzyme-Linked Immunosorbent Assay, ELISA). Moreover, in order to make sure that the results were unbiased, the ELISA experiments were carried out by GenScript with blinded samples.

In contrast to what Strom and Andreasen stated in their Comment, the basis of this study was the general consensus that high molecular weight iron dextran complexes, most of which are no longer marketed, display higher rates and generally more severe hypersensitivity reactions [4,5,6]. Thus, it was evident to start with the investigation of the reactivity of some of the newer intravenous iron products with anti-dextran antibodies. We were not aware of the study by Kreimeier et al. [7] that reports a single clinical case of an antibody-mediated reaction to hydroxyethyl-starch (HES). Nevertheless, despite the fact that carboxymaltose is a branched starch derivative, because of the extensive substitution (hydroxyethyl) in HES, the two compounds are not comparable. Investigation of the reactivity of various intravenous iron preparations, and in particular ferric carboxymaltose, against anti-HES antibodies would be of interest, but is clearly outside the scope of the work described in [1].

I would also like to emphasize that in [1], as well as in all our previous publications on this topic [3,8], we did not make any claim regarding the clinical relevance of these experiments. Conversely, we repeatedly stated that the mechanism of intravenous iron-induced hypersensitivity reactions has not been elucidated yet and that more studies are needed to unravel the mechanism(s). Nevertheless, it is intriguing that a number of recent studies have come to the conclusion that the frequency of hypersensitivity reactions is higher with dextran-based intravenous iron preparations (not only with the old high molecular weight compounds) than with non-dextran containing products [9,10,11,12]. Noteworthy, these reports are in disagreement with the paper mentioned by Strom and Andreasen [6], which, incidentally, was published online more than a month after our final draft was accepted. Taken together, these contrasting reports support the fact that, because of the low incidence of hypersensitivity reactions, as well as the lack of a detailed understanding of their mechanism and of what triggers these reactions, any additional data in this field are valuable.

Finally, in 2008, Crichton et al. [13] *speculated* that Dextran 1 (not Isomaltoside 1000) interacts with anti-dextran antibodies when bound to a polynuclear iron core. Our data *demonstrate* that this is the case for Isomaltoside 1000. Notably, the rationale for the design of Iron isomaltoside 1000 was based on the “theoretically reduced anaphylactogenic potential” of the ligand [14,15]. Thus, the conclusion that a non-immunogenic carbohydrate bound to a polynuclear iron core may form multivalent immune complexes and react with antibodies is important and may be valuable for the design of new intravenous iron preparations.
